# Minimal-Drift Heading Measurement using a MEMS Gyro for Indoor Mobile Robots

**DOI:** 10.3390/s8117287

**Published:** 2008-11-17

**Authors:** Sung Kyung Hong, Sungsu Park

**Affiliations:** Dept. of Aerospace Engineering, Sejong University, Seoul, 143-747, Korea

**Keywords:** MEMS gyro, heading angle, drift, self-identification, threshold filter

## Abstract

To meet the challenges of making low-cost MEMS yaw rate gyros for the precise self-localization of indoor mobile robots, this paper examines a practical and effective method of minimizing drift on the heading angle that relies solely on integration of rate signals from a gyro. The main idea of the proposed approach is consists of two parts; 1) self-identification of calibration coefficients that affects long-term performance, and 2) threshold filter to reject the broadband noise component that affects short-term performance. Experimental results with the proposed phased method applied to Epson XV3500 gyro demonstrate that it effectively yields minimal drift heading angle measurements getting over major error sources in the MEMS gyro output.

## 1. Introduction

Autonomous indoor mobile robots are gaining increasing popularity for the shared use in military and civil applications. Recently aerial or ground robots of various shapes and sizes have been developed for quick detection and observation of circumstances in calamity environments such as indoor fire spots [1, 2]. In an indoor environment, however, weak reception of GPS signals has posed additional challenges to the operation of the robots. Therefore less expensive self-localization with unaided external sensors is one of the most important problems in popularizing indoor mobile robot products. However, trade-offs exist between making less expensive self-localization systems and the quality at which they perform. Relative localizations that utilize low-cost gyroscope (hereafter refer to as a gyro) based on technology referred to as Micro Electro-Mechanical System (MEMS) with odometry sensors have emerged as standalone solutions robust to environment changes [3-5]. However, the performance characteristics associated with these low-cost MEMS gyros are limited by various error sources that affect long-term and short-term performance, such as the bias/scale-factor error and the Angle Random Walk (ARW), respectively [6-9]. Due to the numeric integration process of angular velocity (rate) output of gyros for the angle estimation purposes, gyros are usually required to meet the high performance demands. Otherwise, the accumulated angular error grows considerably over time and provides a fundamental limitation to any angle measurement that relies solely on integration of rate [4-8]. Therefore self-localization of a robot with unaided (without odometer/velocity or GPS or magnetometer aiding) low-cost MEMS gyro is still a challenging problem [10, 11].

To meet the challenges of low-cost MEMS gyros, this paper examines an effective method of minimizing drift on the heading angle that relies solely on integration of rate signal getting over major sources of error. This method can be extended to further reduce the localization error in cooperation with absolute localization method that uses external beacons or landmarks as well as relative one that uses internal odometry sensors [3-5]. The main idea of the proposed approach is consists of two parts. First, during startup, time-varying calibration coefficients of both ‘scale factor error’ and ‘bias’ are simultaneously estimated online and stored in memory. Subsequently, when a gyro measurement is taken, it is compensated with the estimated coefficients. This means that the calibration coefficients that affect long-term performance are updated regularly so that the performance is kept consistent regardless of stochastic aging effects. The second part employed is to threshold the output from the compensated gyro signal when there's no turning motion. That is, the broadband noise components at the gyro output (ARW) which lie under a certain threshold value are filtered out and set to zero. This means that the ARW that affects the short-term performance is partially rejected when there's no turning motion.

An EPSON XV3500 MEMS gyro [12] was selected as the candidate at our lab. It has performance indexes of 18°/hr bias stability, 2.5°/√hr ARW. These specifications say that if we integrate the signals of this gyro for 30 minutes (assumed cleaning robot's operating time) it can be supposed to have the standard deviation of the distribution of the angle drift up to 11°, which provides a fundamental limitation to any angle measurement that relies solely on integration of rate. However, experimental results with the proposed phased method applied to XV3500 demonstrate that it effectively yields minimal-drift angle measurements getting over major error sources that affect both long-term and short-term performance.

This paper is organized as follows. Section 2 describes the performance characteristics for the gyroscope XV3500 through experimental tests. In Section 3, the algorithms for minimal-drift heading angle measurement including online self-calibration with least squares algorithm and threshold filter are presented. In Section 4, the experimental results are provided to demonstrate the effectiveness of the proposed phased method. Concluding remarks are given in Section 5.

## 2. The Performance Characteristics of MEMS Gyros

**Figure 1.**
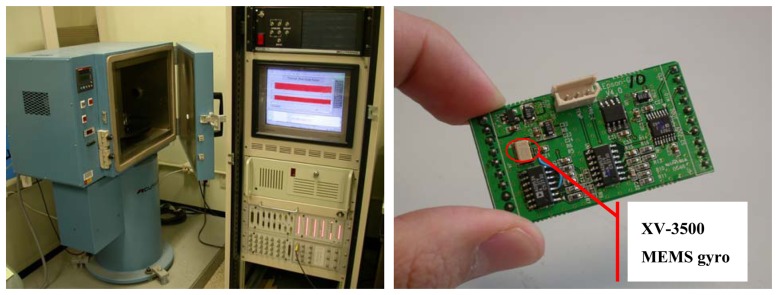
Rate/positioning table and Epson XV3500 gyro module.

The MEMS gyros used in this research are of a quality that is labeled as “automotive grade.” This term is used to describe these sensors because their primary application is in the automotive industry where they are used for active suspension and skid control via rate measurement, not angle measurement [13, 14]. These sensors range in cost from $25 to $1,000 and are expected to drop in price in the future. In this study the rate gyro that was used and tested extensively was the EPSON XV3500 (shown in Figure 1) that costs less than $50. The information provided on the manufacturer supplied data sheets is not normally sufficient for performance analysis. Using methodology similar to that outlined in [14], the performance characteristics for these gyros are evaluated through experimental tests.

We used 10 sets of XV3500 sensors to perform stability tests, rate transfer tests, and thermal/aging tests. Acutronic BD125 (shown in Figure 1), which has a rate resolution of 0.00001°/s, is used as a 1-axis rate table, and the chamber temperature can be varied to investigate temperature dependencies. In the stability tests, bias stability is measured and angle random walk (ARW) is calculated that is a representative index of the gyroscope's short-term performance. In the rate transfer tests, the scale factor error due to its nonlinearity is investigated. The bias changes due to temperature changes and the scale factor changes due to aging effects are measured in the thermal/aging test [8].

### 2.1. Stability Tests

**Figure 2.**
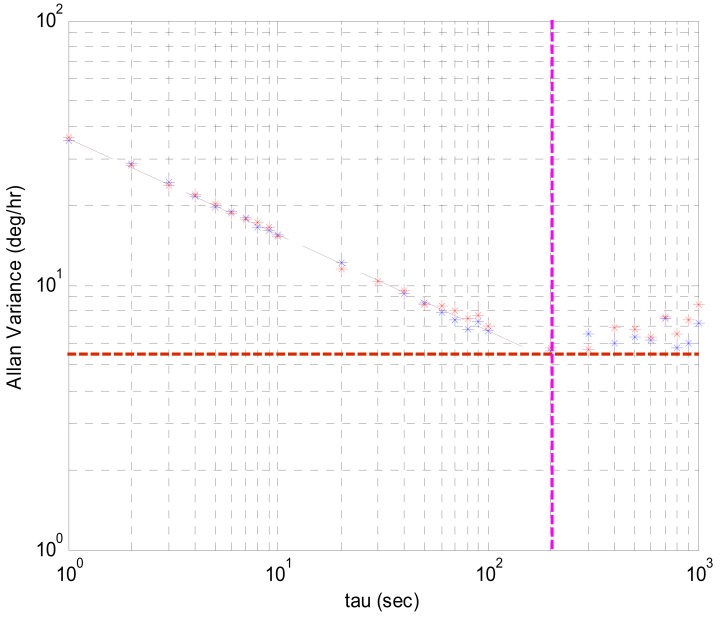
Allan Variance Chart.

Characterization of the stability of the XV3500 gyro was accomplished by constructing and analyzing an Allan-variance chart. Figure 2 shows an Allan-variance chart for a sample of one XV3500s. After two repeated experiments for 10 sets of gyros, averaged ARW defined as the broadband noise component was 2.5°/√hr. This represents the short-term performance limit of XV3500 in that the standard deviation of the angle distribution obtained by integrating rate signals for an hour is 2.5 degrees. And it also shows that the mean bias instability, which is the maximum deviation of the random variation of the bias, is 17.99°/hr. This instability tends to dominate the long-term performance. These levels of uncertainty deteriorate the computational accuracy of the angle measurement and provide a fundamental limitation to any angle measurement that relies solely on integration of rate.

### 2.2. Rate Transfer Tests

**Figure 3.**
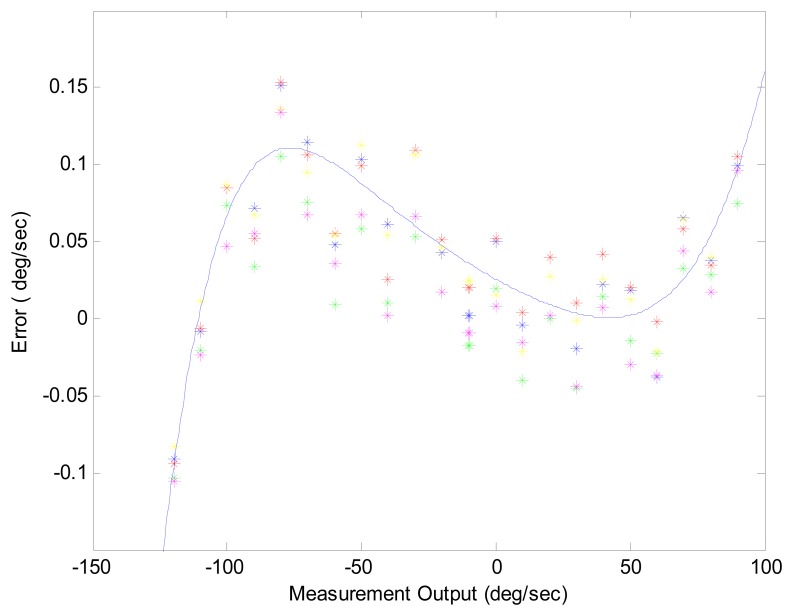
Scale factor error.

Scale factor error is expressed as a ratio of output error to input rate, in parts million (ppm), or as a percentage figure for the lower performance class of sensor like XV3500. To evaluate scale factor error, the gyro is mounted on an accurate rate table. The table is rotated through a series of rates designed to make the errors observable. The experiments involved 22 different rates ranging from -100 to 100deg/s. Figure 3 shows complex error behaviors of one XV3500 gyro. After two repeated experiments for 10 sets of gyros, averaged scale factor error was about 2.53%, which means the angular error is in the area of 9.1 degree after one revolution. This will be a fundamental uncertainty in the result of the angle calculation.

### 2.3. Thermal and Aging Tests

**Figure 4.**
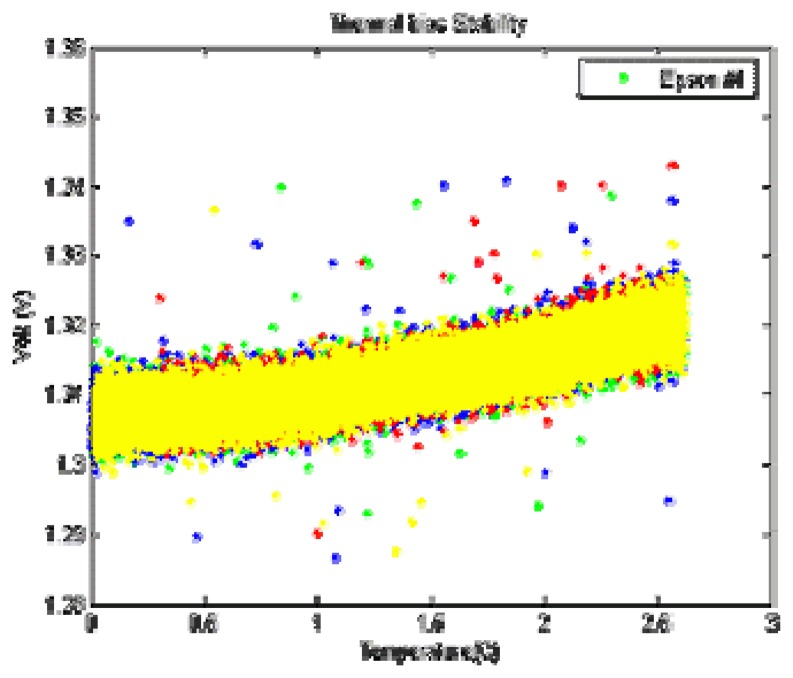
Thermal bias drift.

**Figure 5.**
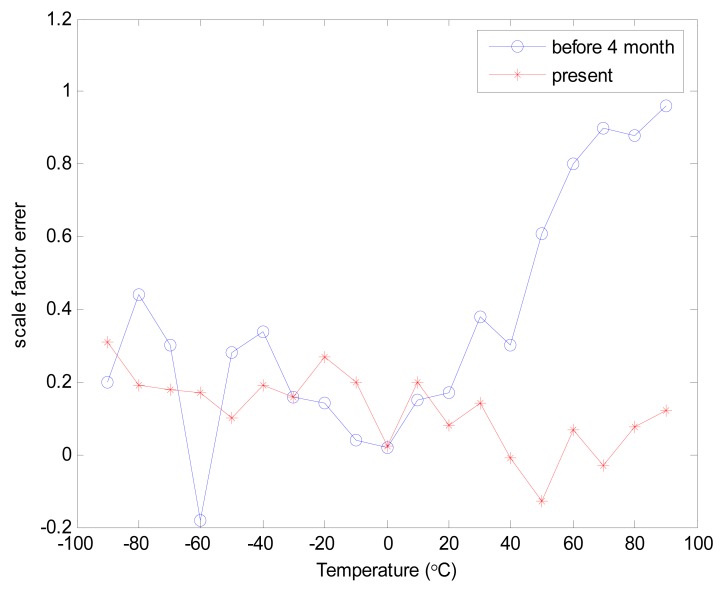
Scale factor changes due to aging.

The gyros were placed in a temperature chamber and the gyro output voltage was monitored. Since the rate gyros were not rotating, slow changes, if any, in the output voltage would be indicative of bias drift. This was repeated for a number of temperatures between 0°C and 25°C. The gyros were allowed to reach thermal equilibrium at each new temperature before data collection. Figure 4 shows the output from a XV3500 that were used during this test. Figure 5 shows that the characteristics of scale factor error have been changed over four months due to aging effects. Even in some case, when a good thermal compensation is provided [8], the variations of characteristic parameters due to aging cannot be predicted or corrected for. Therefore, self-calibration module that updates calibration parameters regularly is essential for commercial systems.

## 3. Minimal-drift Heading Angle Measurement

In Section 2, we found that XV3500 as a low-cost MEMS gyro used here is dominated by three major error sources: scale-factor error (* s *), bias (* b *), and ARW defined as the broadband noise component (* w *). Moreover, it has been shown that *s* and *b* are subject to variations due to environmental changes such as temperature changes and aging. Now we assume the output of the gyro is written

(1) $$r_{m}(t)=(1+s)r(t)+b+w$$

where *r_m_* (*t*) is the measured rate from gyro and *r*(*t*) is the true rate about the sensitive axis.

In such harsh condition, to estimate precise angle with unaided (without odometer/velocity or GPS or magnetometer aiding) low-cost MEMS gyro is a challenging problem. In this Section, we examines an effective method of minimizing drift on the heading angle that relies solely on integration of rate signal getting over major sources of error. The main idea of the proposed approach is consists of two parts: 1) self-identification of calibration coefficients (*s* and *b*) that affects long-term performance, and 2) threshold filter to reject the broadband noise component (*w*) that affects short-term performance.

### 3.1. Self-calibration with Least Squares Algorithm

Our initial focus is on self-calibration of the gyro, that is, to update calibration coefficients (*s* and *b*) that have been changed somehow. During startup phase of an indoor mobile robot, time-varying calibration coefficients of both *s* and *b* are simultaneously estimated and stored in memory. Subsequently, when a gyro measurement is taken, it is compensated with the estimated coefficients. This means that the calibration coefficients that affect long-term performance are updated regularly so that the performance is kept consistent regardless of unpredictable changes due to aging. Ignoring the broadband noise component (*w*) in (1), the output of the gyro can be written

(2) $$r_{m}(t)=(1+s)r(t)+b$$

We will often find it more convenient to express (2) as

(3) $$r(t)=\bar{s}r_{m}(t)+\bar{b}$$

where $\bar{s}=\frac{1}{1+s}$, and. $\bar{b}=\frac{-b}{1+s}$.

**Figure 6.**
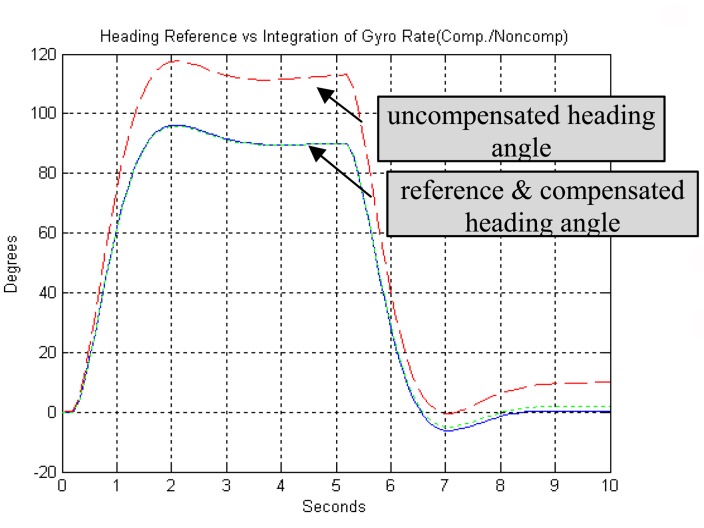
LS algorithm with heading reference.

**Figure 7.**
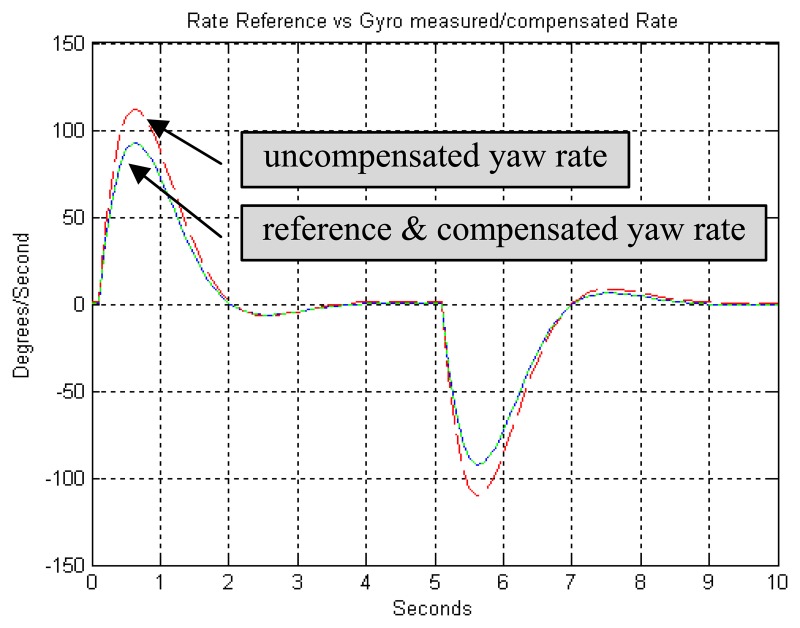
LS algorithm with rate reference

1) Least squares algorithm with heading reference
  The least squares algorithm presented in [8] is introduced to find the calibration coefficients, *s̄* and *b̄* of (3). Consider the discrete-time state equation relating heading and yaw rate
  (4) $$\psi (k+1)=\psi (k)+h(\bar{s}r_{m}(k)+\bar{b}),\;k\geq 0$$
  where *ψ*(*k*+1) is the future heading, *ψ*(*k*) is the initial heading, and *h* is sample period. For any future index *n* > *k* ≥ 0, (4) is rewritten
  (5) $$\psi (k+n)=\psi (k)+h\;\bar{s}\sum_{i=k}^{k+n-1}r_{m}(i)+\;n\;h\;\bar{b}$$
  Taking a number of reference (true) values of heading from the predetermined known motion profile of a robot platform (i.e. typical open-loop controlled motion profile that is assumed to be lifetime identical with precise data set provided by a factory) and measurements from the gyro for increasing *n* and stacking the equations yields the matrix equation:
  (6) z=Gq
  where $z=\left[\begin{array}{c} \psi (k+1)-\psi (k)\\ \psi (k+2)-\psi (k)\\ \vdots \\ \psi (k+n)-\psi (k) \end{array}\right]$, $q=\left[\begin{array}{c} \bar{s}\\ \bar{b} \end{array}\right]$, *G*=[*G*_1_ *G*_2_], $G_{1}=\left[\begin{array}{c} h\;r_{m}(k)\\ h\sum_{i=k}^{k+1}r_{m}(i)\\ \vdots \\ h\sum_{i=k}^{k+n-1}r_{m}(i) \end{array}\right]$, *and* $G_{2}=\left[\begin{array}{c} h\\ 2h\\ \vdots \\ \text{nh} \end{array}\right]$
  Now a least squares estimate of the scale and bias coefficients can be found by solving (6) for *q*
  (7) $$q={\left(G^{T}G\right)}^{-1}G^{T}z$$
  The vector *q* can be found as long as *G^T^G* is nonsingular, meaning that the robot should be changing rate during the calibration.
2) Least squares algorithm with rate reference
  If the reference (true) values are given as angular rate (instead of heading angle) about the predetermined known motion profile of a robot platform, this algorithm is reduced to just direct least squares problem to find *s̄* and *b̄* of (3) that best fit the data (*r_m_*) to true reference data (*r*). The following matrix equation is the result of the least squares analysis and can be used to solve for *s̄* and *b̄* using Kramer's Rule:
  (8) $$\left[\begin{array}{cc} \sum_{i=k}^{n}r_{m}(i) & \sum_{i=k}^{n}{r_{m}}^{2}(i)\\ n & \sum_{i=k}^{n}r_{m}(i) \end{array}\right]\left\{\begin{array}{c} \bar{s}\\ \bar{b} \end{array}\right\}=\left\{\begin{array}{c} \sum_{i=k}^{n}r(i)r_{m}(i)\\ \sum_{i=k}^{n}r(i) \end{array}\right\}$$
  Now a least squares estimate of *s̄* and *b̄* can be found by solving (8) for *q*
  (9) $$q={\tilde{G}}^{-1}\tilde{z}$$
  where $\tilde{G}=\left[\begin{array}{cc} \sum_{i=k}^{n}r_{m}(i) & \sum_{i=k}^{n}{r_{m}}^{2}(i)\\ n & \sum_{i=k}^{n}r_{m}(i) \end{array}\right]$, *and* $\tilde{z}=\left\{\begin{array}{c} \sum_{i=k}^{n}r(i)r_{m}(i)\\ \sum_{i=k}^{n}r(i) \end{array}\right\}$The vector *q* can be found as long as *G˜* is nonsingular, meaning that the robot should be changing rate during the calibration.
3) Evaluation
  To evaluate the performance of this algorithm, some simulations are done with heading reference and rate reference data for some specified motion profiles. For all simulations, gyro scale and bias factors were set to *s*= -10 % and *b*= 0.1 *rad*/*sec*, corresponding to coefficient value of *s̄* =1.111, *b̄* =-0.111. Figure 6 shows the discrepancies between reference heading and gyro heading (integration of rate). Figure 7 shows the discrepancies between reference rate and gyro rate. The proposed least squares algorithm with heading reference found the coefficients of *s* (=-9.52%) and *b* (=0.08 *rad*/*sec*) with error of 4.8% and 20.26%, respectively. On the other hand, least squares algorithm with rate reference showed 0% error both *s* and *b*. Subsequently, when gyro measurements are compensated with the estimated coefficients, they show almost identical to reference data as shown in Figures 6 and 7.

### 3.2. Threshold Filter

If the scale factor error (s) and bias (b) of the gyro are identified and compensated with the self-calibration procedures described in the of previous section, the output of the gyro can be written as

(10) $$r_{m}(t)=r(t)+d(t)+w$$

where *d* is the residual uncompensated error of the scale factor and bias. The dominant part of error *d* comes from the stochastic bias which is the unpredictable time-varying drift of bias, and the remaining part is the effect of uncompensated scale factor error which can be included as one source of the stochastic bias. Typically, *d* is modeled as a random walk as following.

(11) $$\dot{d}=n$$

where *n* is zero-mean white noise with the spectral density *Q* which can be determined by analyzing the gyro output.

**Figure 8.**
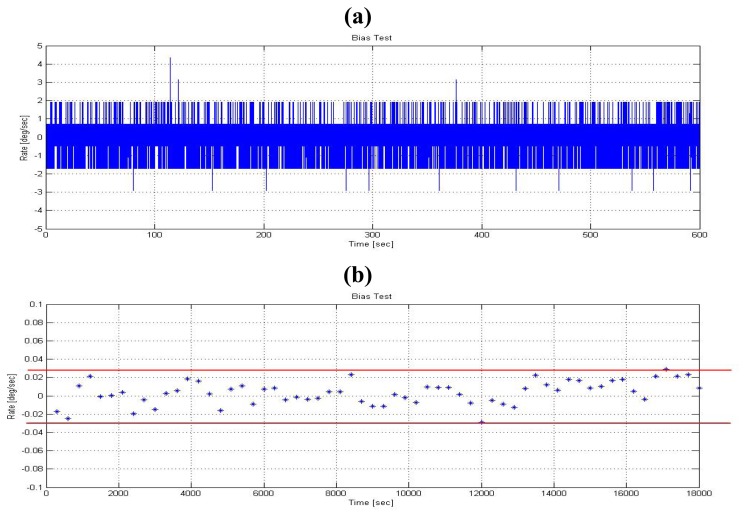
Typical output of EPSON XV3500 (a) broad band noise(*w*), (b) residual error (*d*)

Figure 8(a) shows a typical output of EPSON XV3500 gyro. Note that the peak-to-peak noise is about 2 *deg*/*sec*, which is dominated by the broadband noise (*w*). The residual error (*d*) is shown in Figure 8(b), which is obtained by averaging the gyro output based on an averaging time of 5 min. after collecting a long term sequence of data. In this figure, the upper and lower bounds of the uncertainty are also plotted. These uncertainty bounds limit the minimum detectable signal of angular rate, and thus limit the accuracy of the heading angle calculated by integrating angular rate.

Most of currently employed signal processing methodologies utilize Kalman filtering techniques to reduce the effect of ARW and to improve the estimation accuracy [15, 16]. Unfortunately, the convergence of these methods (the time to remove the effect of the ARW and to provide an accurate estimate) during the alignment processes can take up to 15 min. In addition to their time-consuming algorithms, these techniques are complex to design and require a considerable effort to achieve real-time processing. Although some modern techniques have been introduced to reduce the noise level and some promising simulation results have been presented, real-time implementation with an actual MEMS gyro has not yet been reported.

For a low-cost and simple, but an effective methodology, we implemented threshold filter that the values in the rate gyro signal which lie under a certain threshold (*r_thres_*) are filtered out and set to zero.

(12) $$\begin{array}{cc} \text{if}\;\left|r_{m}\right|<r_{\text{thres}}, & \text{then}\;r_{m}=0 \end{array}($$

This means that the effects of ARW are partially eliminated when there's no turning motion. This filters out noise but of course also eliminates the gyro's capability to sense very slow turns (with the current threshold setting, *r_thres_* = 0.3 deg/s). This has only limited significance, however, since the mobile robot which is the application target of this study can only move with a certain minimum angular velocity (≫1 deg/s). Of course, slow changes in direction during “pure” translation are also ignored but this is not supposed to be a major error source in the vehicle's odometry.

## 4. Experimental Results

### 4.1. Experimental Setup

**Figure 9.**
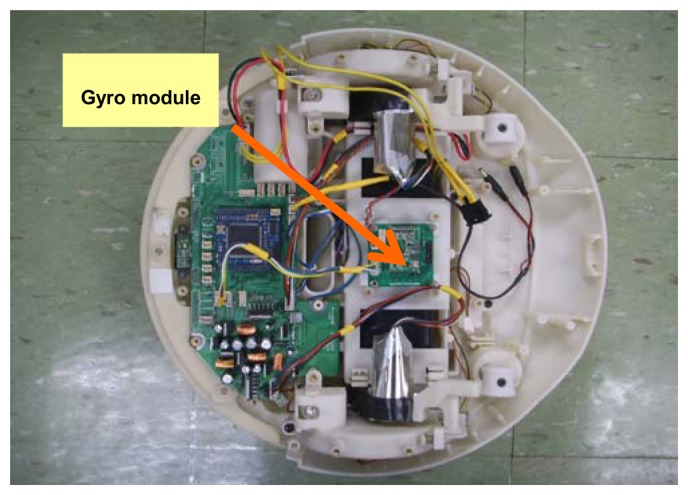
Robot Platform.

The technique proposed in this paper was tested using an Epson XV3500, a low-cost MEMS gyro with 17.99 deg/h bias drift, and 2.5°/√hr ARW. It is mounted on the robot platform that is a self-made conventional two-wheel differential drive with two casters (Figure 9). The robot was tested on a preprogrammed trajectory for 160 sec and self-calibration period are given for 20 sec. We used the Hawk Digital system from Motion Analysis, Inc. to obtain the ground truth data of the robot heading. The robot traveled at a maximum speed of 2.0 m/s, and the reference data and XV3500 gyro measurements were acquired at sampling rates of 20 and 100 Hz, respectively.

### 4.2. Results

**Figure 10.**
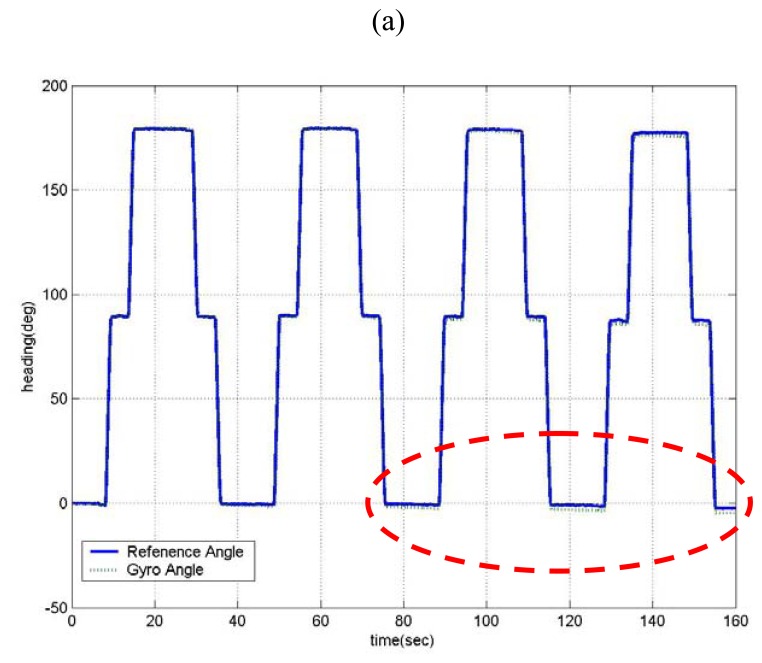

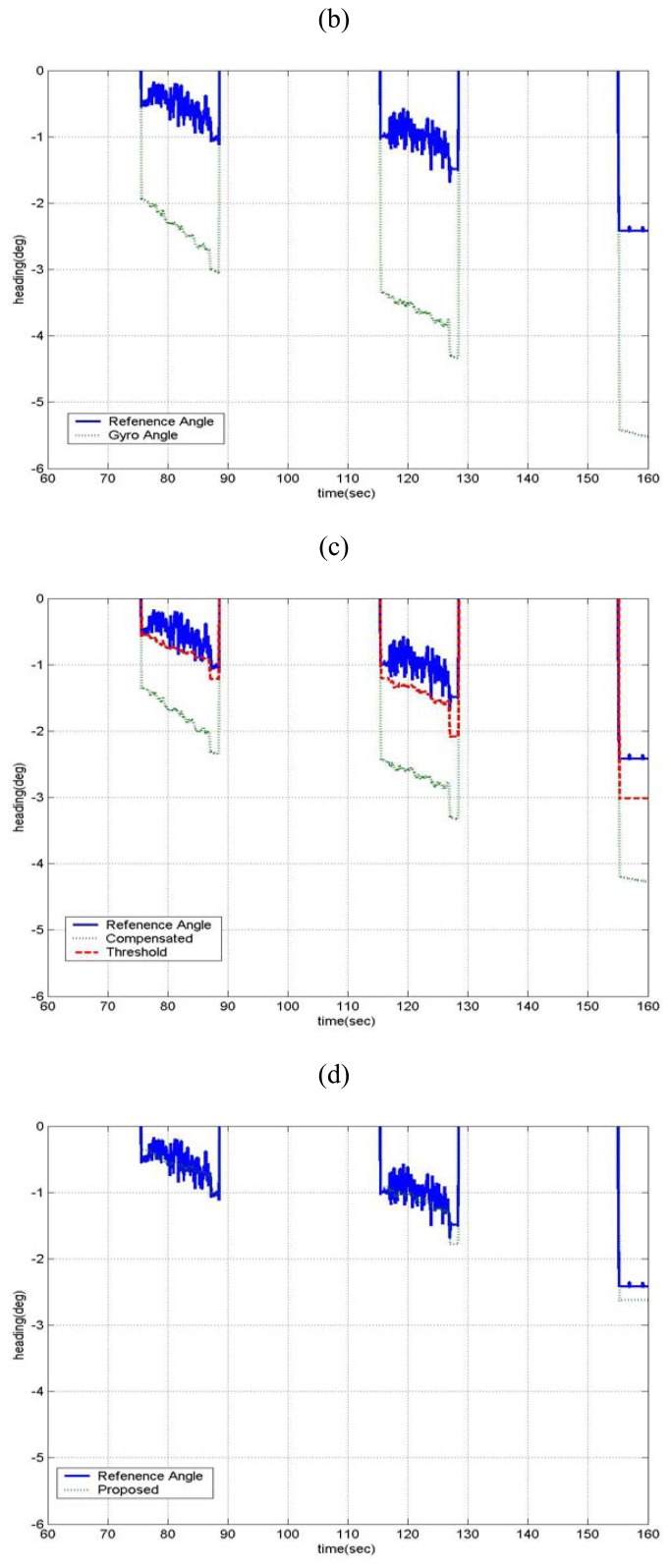
Experimental results for each method.

The heading angles correspond to preprogrammed trajectory is shown in Figure 10(a). The angle error is the mean absolute value of error between the true and the estimated value. From Figure 10(b), that is exaggerated some part (the circle region of 60 sec-160 sec) of Figure 10(a), it can be seen that the error of gyro heading (pure integration of rate signal) is growing over time. It shows a fundamental limitation to any angle measurement that relies solely on pure integration of rate signal. On the other hand, after self-calibration (estimated values of *s* and *b* are 3.73% and 0.75 deg/s) or threshold filtering, the mean error has been reduced by 41% and 53% respectively, compared with pure integration result [shown in Figure 10(c)]. This improvement, of course, is due to proper handling of major error sources that affects long-term or short-term performance individually. However, both results show some drift over time. Based on this testing and analysis, it was concluded that all the error sources (bias/scale-factor error and the ARW) that affect long-term and short-term performance have to be considered at the same time. From Figure 10(d), it can be seen that the heading angle of the proposed method (both self-calibration and threshold filtering) almost follows the true heading showing little drift at all time, showing 64% error reduction. This is considered to be quite good performance for automotive grades. However, it should be noted that perfect drift free heading is possible only when ARW that exists even in the turning motion is rejected. In our proposed method, threshold filter can reject noise components only when no rotation is applied. All the results are summarized in Table 1.

## 5. Conclusions

To meet the challenges of making low-cost MEMS gyros for the precise self-localization of indoor mobile robots, this paper examines an effective and simple method of minimizing drift on the heading angle that relies solely on integration of rate signals from a gyro. The main idea of the proposed approach is consists of two parts; 1) self-identification of calibration coefficients that affects long-term performance, and 2) threshold filter to reject the broadband noise component that affects short-term performance. Experimental results with the proposed phased method applied to Epson XV3500 gyro demonstrate that it effectively yields minimal drift heading angle measurements getting over major error sources in the gyro output. We suggest that the MEMS gyro can be used in a wide range of mobile robotic applications, both as a “global reference” but also to help odometry, with extension of proposed method to further reduce the localization error.

## Figures and Tables

**Table 1.** Mean heading angle error for each method (deg)

	**pure integration**	**self-calibration**	**Threshold filter**	**proposed**
**Mean error**	1.6675	1.1511	0.7768	0.5985
**Standard dev.**	1.5075	1.1675	1.2653	1.2998
